# Strangulated transomental hernia causing small bowel obstruction in a virgin abdomen

**DOI:** 10.1093/jscr/rjag586

**Published:** 2026-07-15

**Authors:** Unaiza Erum, Omer Bin Khalid Jamil, Reem Shahid, Ayesha Noman, Shahrukh Imtiaz, Zahra Zafar, Mahnoor Babar

**Affiliations:** Department of General Surgery, Foundation University, Defence Avenue, DHA Phase-I, Islamabad Capital Territory 44000, Pakistan; Department of General Surgery, Dow University of Health Sciences, Baba-e-Urdu Road, Karachi 74200, Pakistan; Department of General Surgery, Foundation University, Defence Avenue, DHA Phase-I, Islamabad Capital Territory 44000, Pakistan; Department of General Surgery, Dow University of Health Sciences, Baba-e-Urdu Road, Karachi 74200, Pakistan; Department of General Surgery, Foundation University, Defence Avenue, DHA Phase-I, Islamabad Capital Territory 44000, Pakistan; Department of General Surgery, Foundation University, Defence Avenue, DHA Phase-I, Islamabad Capital Territory 44000, Pakistan; Department of General Surgery, Foundation University, Defence Avenue, DHA Phase-I, Islamabad Capital Territory 44000, Pakistan

**Keywords:** transomental hernia, internal hernia, virgin abdomen, closed-loop obstruction, strangulated bowel, small bowel obstruction

## Abstract

Transomental hernia is a rare cause of small bowel obstruction and may be missed before surgery, particularly in a virgin abdomen. We report a 75-year-old woman with no previous abdominal operation who presented with 4 days of abdominal pain, vomiting, distension, constipation, and reduced urine output. She had leukocytosis and acute kidney injury. Because of renal dysfunction, computed tomography was performed without intravenous contrast. It showed dilated ileal loops, a pelvic transition point, mild ascites, and twisting of mesenteric vessels at the transition point, suggestive of volvulus or closed-loop obstruction. Internal hernia was not diagnosed preoperatively. Emergency laparotomy revealed ileum strangulated through a 3 cm greater omental defect. Approximately 120 cm of gangrenous ileum was resected, the defect was excised, and single-layer extramucosal hand-sewn end-to-end anastomosis was performed. The case emphasizes that closed-loop obstruction in a virgin abdomen should prompt early consideration of internal hernia.

## Introduction

Small bowel obstruction is common in emergency general surgery, but the cause may be less predictable in a patient without previous abdominal surgery. In this setting, postoperative adhesions are less likely, and alternative causes such as malignancy, external hernia, inflammatory stricture, volvulus, bezoar, congenital band, and internal hernia must be considered [1–3].

Internal hernias account for ~0.6%–5.8% of small bowel obstruction cases, but trapped bowel may rapidly progress to venous congestion, ischemia, and gangrene [4–6]. Transomental hernia is particularly rare. In older patients, the omental defect may be acquired, possibly due to thinning or degeneration of the greater omentum [4, 7].

Preoperative diagnosis can be difficult, especially when computed tomography (CT) is performed without intravenous contrast. We report a strangulated transomental hernia in a virgin abdomen, diagnosed at laparotomy after non-contrast CT suggested closed-loop small bowel obstruction.

## Case report

A 75-year-old woman presented to the emergency department with 4 days of colicky abdominal pain, repeated vomiting, progressive abdominal distension, constipation, and reduced urine output for 2 days. Her medical history included hypertension, chronic obstructive pulmonary disease, and treated pulmonary tuberculosis 20 years earlier. She had no history of abdominal surgery, abdominal trauma, or previous bowel obstruction.

On arrival, she appeared ill but was conscious and oriented. Her pulse was 108 beats/min, blood pressure 150/70 mmHg, oxygen saturation 94% on room air, and Glasgow Coma Scale score 15/15. The abdomen was distended, with mild generalized tenderness and guarding. Bowel sounds were audible. Digital rectal examination revealed hard stool in the rectum with preserved sphincter tone.

Blood tests showed hemoglobin 10.4 g/dL, leukocytosis of ~13 000/mm^3^, raised blood urea, and acute kidney injury with serum creatinine ~4.0 mg/dL. Arterial blood gas analysis showed respiratory alkalosis, with pH 7.52 and pCO₂ 29.2 mmHg. Abdominal ultrasonography did not identify the cause of obstruction.

Because of acute kidney injury, CT was performed without intravenous contrast. It showed dilated ileal loops with multiple air-fluid levels, reaching ~4 cm in diameter, and a transition point in the mid-pelvis at S1 level. A small bowel feces sign was seen in one loop. Deep in the pelvis, there was a closed loop of dilated bowel with twisting of mesenteric vessels at the transition point, suggestive of volvulus or closed-loop obstruction. Mild abdominopelvic ascites was present. No mass was seen at the transition point, and the ileocecal junction appeared normal. The radiological impression was small bowel obstruction, possibly from adhesions or phytobezoar, with concern for a closed-loop component. Internal herniation was not diagnosed before surgery (Figs 1–3).

**Figure 1:**
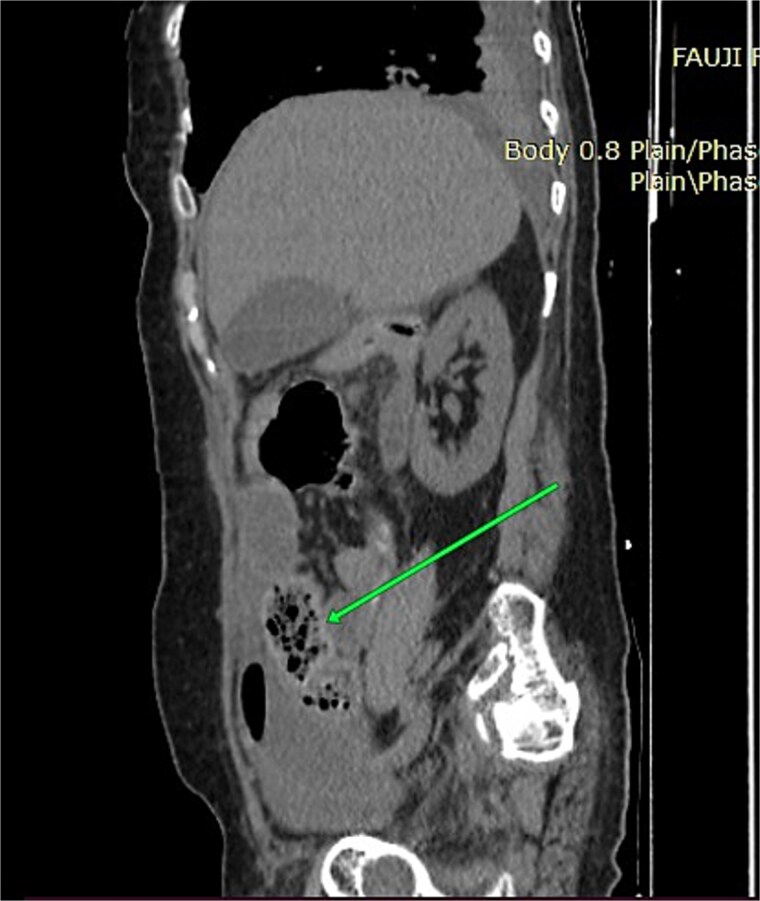
Sagittal non-contrast CT image showing dilated ileal loops with mottled intraluminal gas, representing the small bowel feces sign.

**Figure 2:**
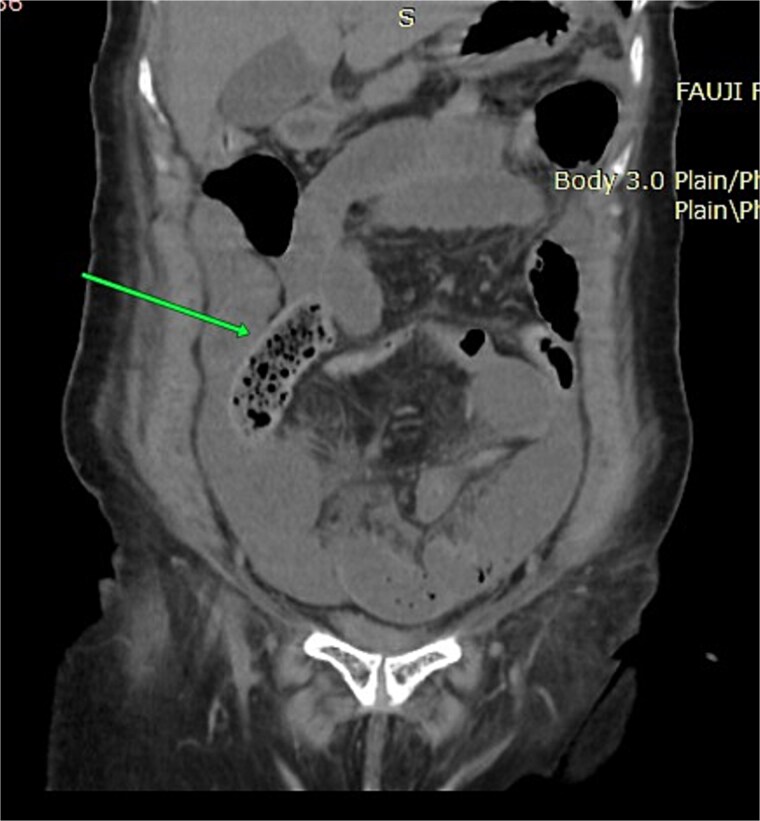
Coronal non-contrast CT image showing clustered dilated ileal loops in the lower abdomen and pelvis, with narrowing toward the transition point.

**Figure 3:**
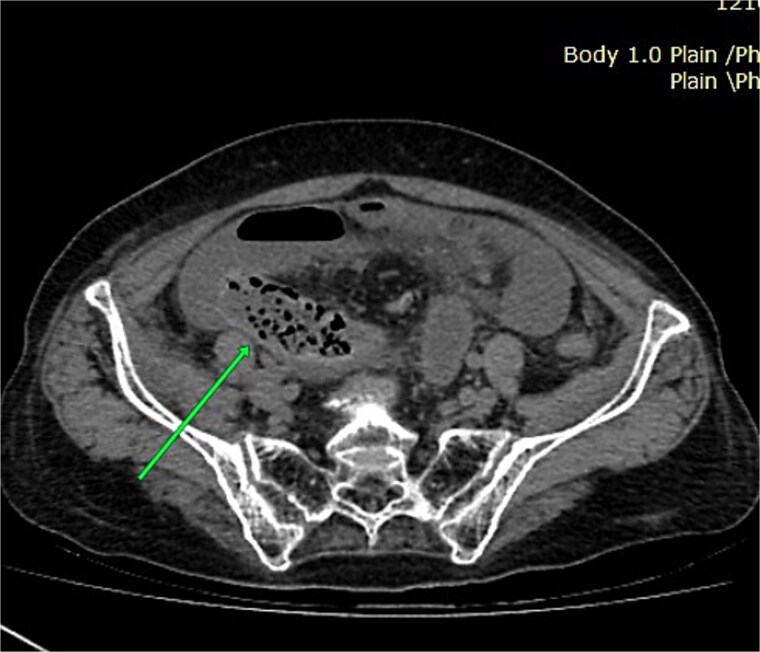
Axial non-contrast CT image showing a curved pelvic ileal loop with twisting of adjacent mesenteric vessels at the transition point.

Because of clinical deterioration, renal impairment, and CT concern for strangulation, emergency exploratory laparotomy was performed through a midline incision. At laparotomy, distal ileum was trapped through a 3 cm defect in the greater omentum, confirming a transomental hernia. Approximately 120 cm of ileum, beginning ~1.5 ft proximal to the ileocecal junction, was gangrenous and did not regain viability after reduction and warm saline application. The dusky adjacent omentum was excised with division of the defect. The remaining bowel was viable, with no gross peritoneal contamination.

The gangrenous ileum was resected, and a single-layer extramucosal hand-sewn end-to-end anastomosis was performed. A diverting stoma was not created because the bowel ends were healthy, there was no contamination, and she remained hemodynamically acceptable intraoperatively (Figs 4 and 5). Postoperatively, she required intensive care, mechanical ventilation, and vasopressor support. Her course was complicated by septic shock, worsening acute kidney injury, altered sensorium, failed ventilator weaning, electrolyte imbalance, respiratory failure and progressive multi-organ dysfunction. She died on postoperative day seven.

**Figure 4:**
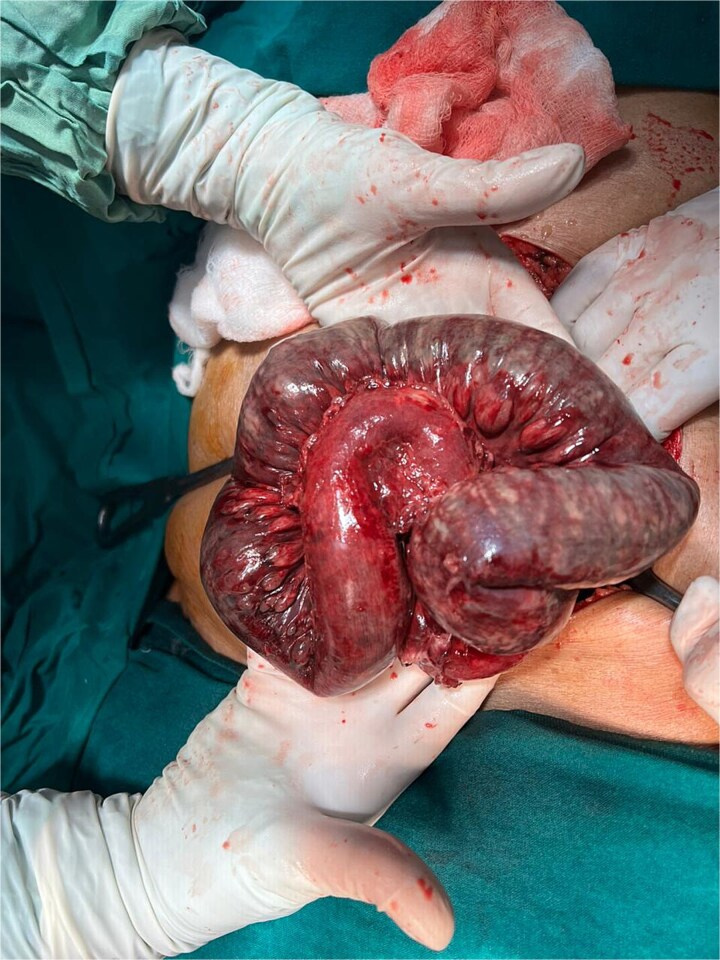
Intraoperative photograph showing the distended, congested ileal loop after delivery through the midline laparotomy incision.

**Figure 5:**
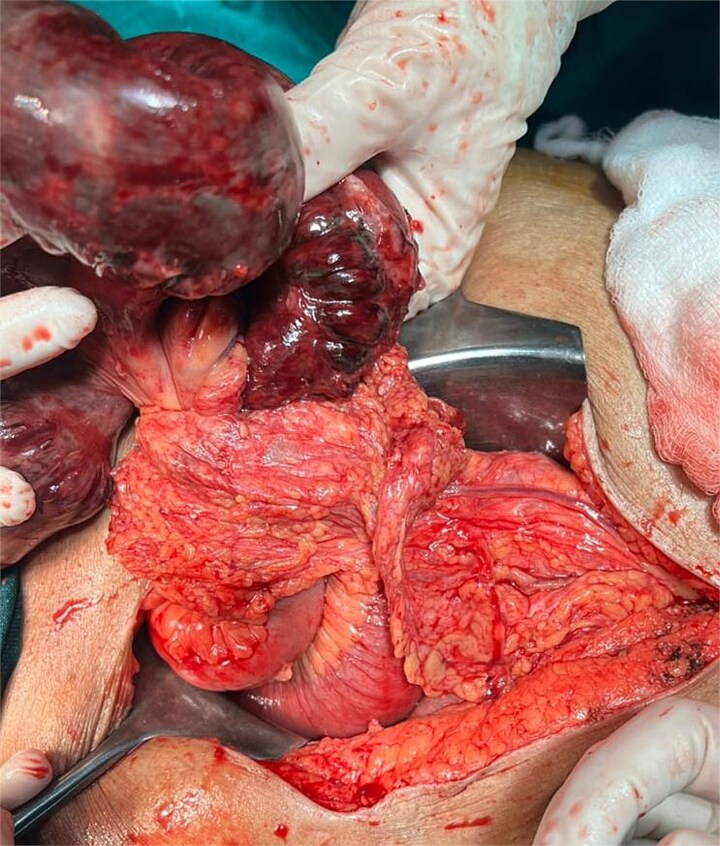
Intraoperative photograph showing gangrenous ileum lying adjacent to the greater omental defect through which the bowel had herniated.

## Discussion

Transomental hernia is an uncommon type of internal hernia in which bowel passes through a defect in the greater omentum. Such defects may be congenital, traumatic, postoperative, inflammatory, or age-related. In an elderly patient without previous laparotomy, thinning or degeneration of the omentum is a plausible cause, although this cannot usually be proven at operation [4, 7].

The danger lies in the narrow opening through which bowel becomes trapped. Venous congestion may progress quickly to ischemia and gangrene, yet the patient may present with non-specific features of small bowel obstruction. In a virgin abdomen, this can be misleading because clinicians often look first for malignancy, external hernia, bezoar, volvulus, or congenital bands rather than a transomental defect.

In this case, ultrasound did not contribute to the diagnosis. CT showed obstruction, a transition point, small bowel feces sign, mild ascites, and a closed-loop configuration with mesenteric twisting. However, the omental defect itself was not identified, and the initial radiological impression favored adhesions or phytobezoar. This difference between the scan report and operative finding is important because CT may show the consequences of internal herniation more clearly than the actual defect, especially when intravenous contrast cannot be given. In a virgin abdomen, the combination of closed-loop obstruction, mesenteric twisting, and ascites should raise internal hernia high in the differential diagnosis [5, 6, 8].

Management depends on early operation when strangulation is suspected. The operative aims are to reduce the trapped bowel, assess viability, resect non-viable bowel, and eliminate the hernial opening. In our patient, the gangrenous ileum was resected, the omental defect excised, and primary anastomosis performed. Diversion was not performed because the bowel ends were healthy, there was no gross contamination, and the patient remained hemodynamically acceptable during surgery.

The patient presented late, with dehydration, acute kidney injury, comorbid lung disease, and a long segment of gangrenous bowel. Postoperative sepsis, renal dysfunction, ventilator dependence, and multi-organ dysfunction led to fatal deterioration. In small bowel obstruction in a virgin abdomen, a closed-loop pattern with mesenteric twisting should prompt early operative consideration, even when the exact cause is not defined preoperatively.
